# Synthesis, Structure, and Properties of 2D Lanthanide(III) Coordination Polymers Constructed from Cyclotriphosphazene-Functionlized Hexacarboxylate Ligand

**DOI:** 10.3390/molecules29235602

**Published:** 2024-11-27

**Authors:** Qi Jia, Yicheng Yao, Xiaoming Zhu, Juntao Wang, Zeyu Li, Liudi Ji, Peng Hu

**Affiliations:** 1 School of Pharmacy, Hubei University of Science and Technology, Xianning 437100, China; jq_958@163.com (Q.J.); aoieng2000@163.com (Y.Y.); 2 Hubei Key Laboratory of Radiation Chemistry and Functional Materials, School of Nuclear Technology and Chemistry & Biology, Hubei University of Science and Technology, Xianning 437100, China; zhuxiaoming@mail.hbust.com.cn (X.Z.); juntao-wang@hotmail.com (J.W.); lizeyu725@163.com (Z.L.)

**Keywords:** coordination polymer, cyclotriphosphazene, lanthanide, magnetism, luminescence

## Abstract

The design and synthesis of novel lanthanide-based coordination polymers (Ln-CPs) from flexible organic ligands is still attractive and challenging. In this work, two isostructural Ln-CPs with a unique 2D network, namely, [Ln_2_(H_3_L)_2_(DMF)]]_n_ (Ln = Dy for **1**, Tb for **2**) based on a flexible polycarboxylic acid ligand hexakis(4-carboxylato-phenoxy)cyclotriphosphazene (H_6_L), have been solvothermally synthesized and structurally characterized. Significantly, it is the first observation of polycarboxylic acid ligands participating in coordination in the construction of coordination polymers in the form of semi-deprotonation. Magnetic measurements showed the presence of field-induced slow magnetic relaxation in complex **1**. The luminescence property of **2** had been studied in the solid state at room temperature.

## 1. Introduction

The design and synthesis of novel coordination polymers have garnered significant attention, not only due to their intriguing structures but also because of their noteworthy physical properties and potential applications in magnetism, luminescence, catalysis, and so on [1,2]. In contrast to the well-established transition metals, CPs, lanthanides are virtually underdeveloped owing to some intrinsic characteristics of lanthanide ions such as the high and variable coordination numbers as well as the small energy difference among various coordination geometries [3,4].

Based on the large-spin multiplicity of the spin ground state and the intrinsic magnetic anisotropy, lanthanide ions, particularly dysprosium ions, have long been regarded as the best candidates for the construction of single-molecule magnets for their promising applications in spintronics, high-density magnetic storage, and quantum computing [5,6,7]. In addition, Ln-CPs are well-known candidates showing enhanced photoluminescence characteristics due to f-f transition [8,9]. The above is especially true for Eu(III) and Tb(III) compounds. Therefore, there is an exciting benefit to constructing novel magnetic or luminescent materials consisting of lanthanide ions and appropriate functionalized polycarboxylic acid ligands. As far as we know, various rigid aromatic multicarboxylates have been widely employed in the construction of structurally diverse CPs; however, limited lanthanide-based CPs (Ln-CPs) have been reported when examining flexible functionalized organic ligands [10,11,12].

**Scheme 1:**
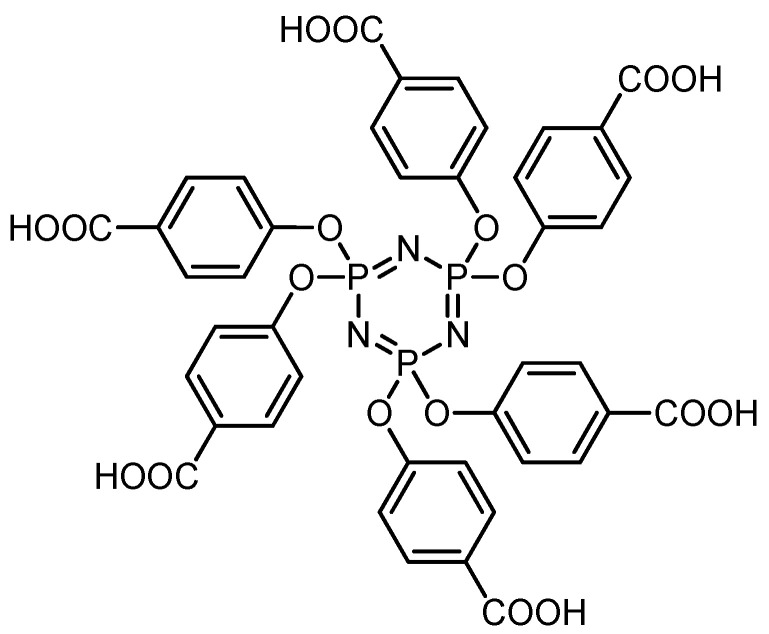
The structure of hexacarboxylic acid ligand H_6_L used in this work.

As a flexible cyclotriphosphazene-functionalized hexacarboxylate ligand, hexakis(4-carboxylatophenoxy) cyclotriphosphazene (H_6_L, Scheme 1) decorated with six free-swinging peripheral arms can develop versatile coordination modes due to the flexible connector between the central cyclotriphosphazene scaffold and the ambient benzoate. Generally, lanthanide ions prefer binding carboxylate oxygen atoms according to the HSAB (hard and soft acid and base) principle, which may increase the chances of successful construction of the Ln-CPs. Furthermore, the easily prepared H_6_L ligand has been applied to build a number of coordination polymers with various topological structures, such as 2D layer network {[Eu_2_(L)(H_2_O)_4_](H_2_O)_4_(DMF)_2_}_n_ [13], 3D open frameworks {[Ho_4_L_2_(CH_3_COO)(H_2_O)]·(CH_3_)_2_NH_2_·20DMA}_n_ [14], and several transition metals and alkaline earth-based coordination polymers [15,16,17,18,19], exhibiting remarkable and inconstant coordination ability. However, without exception, all six carboxylic acid groups in the ligand were deprotonated and participated in the coordination when constructing these coordination polymers. This limits the structural diversification and functionalization of coordination polymers.

Taking into account all of the aforementioned considerations, the excellent flexible ligand H_6_L based on the fully substituted cyclotriphosphazene inorganic scaffold was selected specifically to investigate the role of reaction conditions in the structural control of Ln-CPs. Herein, two novel isostructural two-dimensional Ln-CPs, [Dy_2_(H_3_L)_2_(DMF)]]_n_ (**1**) and [Tb_2_(H_3_L)_2_(DMF)]]_n_ (**2**), were solvothermally synthesized under the condition of strong acidity. Their crystal structure and magnetic and luminescence properties were described in detail. Interestingly, for the first time, it was observed that polycarboxylic acid ligands participated in the construction of coordination polymers with semi-deprotonation.

## 2. Results and Discussion

### 2.1. Synthesis and Characterization 

Two novel 2D Ln-CPs were successfully fabricated by the solvothermal reaction of Ln(NO_3_)_3_·6H_2_O (Ln = Dy, Tb), including H_6_L, a concentrated nitric acid with rated molar ratio in a mixed solvent of DMF/H_2_O (*v*/*v*, 4 mL/1 mL) at 130 °C for 72 h, resulting in the formation of block-shaped colorless crystals. It is worth noting the importance of the amount of concentrated nitric acid for the synthesis of the Ln-CP crystalline materials. A missing or excessive amount of nitric acid during synthesis can contribute to the creation of materials poorly suited for single-crystal X-ray diffraction analysis. In addition, when a small amount of concentrated nitric acid was added to adjust the pH to 2–3, another reported two-dimensional coordination polymer was obtained with all six carboxyl groups in the structure being deprotonated [13]. These results indicate that nitric acid here may play an important role in regulating the deprotonation of ligands and the kinetics of crystal nucleation and growth [20,21,22,23]. 

The structures were characterized by single-crystal X-ray diffraction, and the phase purity was confirmed by good consistency between the measured X-ray diffraction patterns and the theoretical simulated ones based on crystallographic data (Figure S1). The IR spectra reveal signature absorption bands mainly ascribed to the asymmetric (*v*_as_ ca. 1600 and 1536 cm^−1^) and symmetric (*v*_as_ ca. 1423 cm^−1^) stretching oscillations of the carboxylate groups. A strong absorption peak of –COOH near 1699 cm^−1^ is also observed, suggesting that some of the carboxylic groups in the organic moieties of complexes **1**–**2** are not deprotonated, which is consistent with the analysis of X-ray diffraction (Figure S2). The TGA of **1**–**2** under a nitrogen atmosphere displays that there is no weight loss before 390 °C, indicating no free guest molecules or coordinated molecules with low boiling points in the whole molecular system, and these results are in agreement with the crystal structures. Above 390 °C, the apparent weight loss is due to the collapse of the whole framework. The results indicate that they have good thermal stability.

### 2.2. Structural Characterization

**Figure 1:**
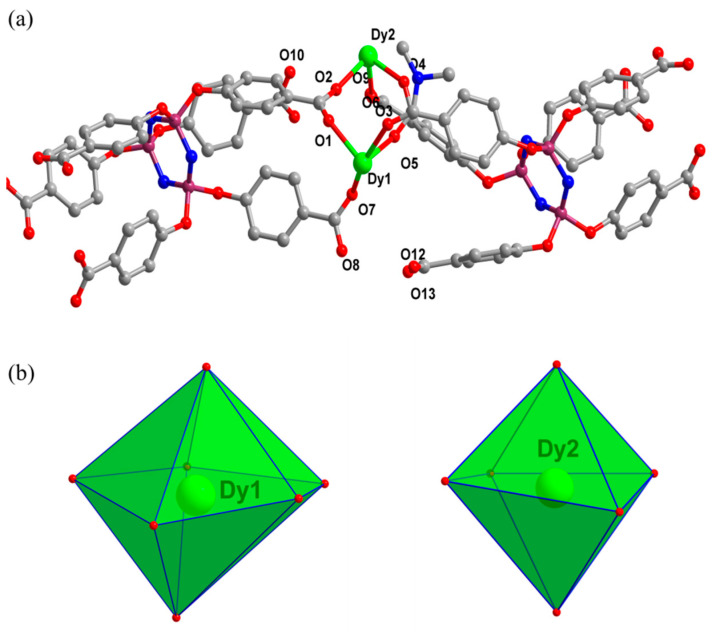
(**a**) The perspective view of the asymmetric unit of compound **1** with partial atoms labeled. Hydrogen atoms have been omitted for clarity. (**b**) The coordination geometry of the Dy1 ion (**left**) and Dy2 (**right**) ion in compound **1**. Color code: Dy^III^, bright green; O, red; N, blue; P, plum; C, gray.

Both complexes **1**–**2** are isostructural as analyzed by single-crystal and powder X-ray diffraction (Table S1, Figure S1). Herein, the structures are described in detail only for complex **1** as an exception. Single-crystal X-ray diffraction analysis shows that complex **1** crystallizes in the triclinic space group *P*2_1_/c, with an asymmetric unit composed of two crystallographically independent Dy^III^ ions, two semi-protonated hexacarboxylate ligands H_3_L, and one coordinated DMF solvent molecule (Figure 1a). The two Dy^III^ centers show different kinds of coordination modes. As shown in Figure 1b, the seven coordinated Dy1 ions are connected with six O atoms from five different carboxylate groups of four distinct partially deprotonated ligands H_3_L and one from the coordinated DMF molecules, resulting in a distorted decahedron geometry, while the six coordinated Dy2 ions feature a distorted octahedral geometry occupied by six O atoms from six different carboxylate groups of five distinct aforementioned ligands. The Dy1 and Dy2 ions are connected by two syn–syn *μ*_2_ chelating carboxyls into a dinuclear structure. The bond distance of Dy–O ranges from 2.210(3) to 2.440(3) Å, which is quite comparable to the ones reported for carboxylate-bridged dysprosium complexes [24,25,26]. The distances of adjacent Dy1···Dy1 and Dy2···Dy2 are 8.926(15) and 9.258(15) Å, respectively.

**Figure 2:**
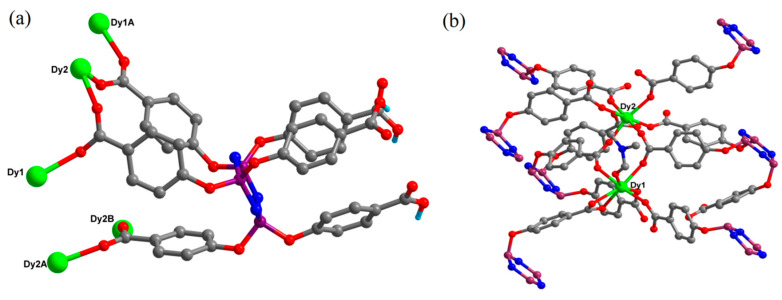
The connection mode of the semi-protonated hexacarboxylic acid ligand (**a**) and dinuclear unit (**b**) of compound **1**. Asymmetry code: A, x, 1/2 − y, −1/2 + z; B, 1 − x, −1/2 + y, 3/2 − z. Color code: Dy^III^, bright green; O, red; N, blue; P, plum; C, gray; H, sky blue.

Concerning the six-armed organic linker, three of the six 4-carboxylato-phenoxy substituents are dispersed on either side of the central cyclotriphosphazene ring, which is almost planar. The average P-N bond length is 1.5823 Å, and the average N–P–N and P–N–P angles are 116.94° and 121.87°, respectively, which are very similar to those previously reported for cyclotriphosphazene derivatives [13,14,15,16]. Interestingly, only one side of the carboxylic groups is deprotonated and participates in coordination with Dy^III^ ions; the other side is on the shelf (Figure 2a). As far as we know, such semi-deprotonated coordination polymers constructed with polycarboxylic acid ligands are first observed here, and harsh strong acid synthesis conditions may be the main reason for this unusual configuration. A large number of flexible free carboxylic groups remained at both ends of the structure providing the possibility for further functionalization. Moreover, it is noteworthy that neither the nitrogen atoms nor the oxygen atoms connected to phosphorus atoms on the cyclotriphosphazene ring participate in the coordination with the metal centers. The deprotonated carboxylic groups employ two different coordination modes that can be separately summarized as the chelating mode (*μ*_1_: η^1^η^1^) and the bridging mode (*μ*_2_: η^1^η^1^) (Figure S4), while the two bridged carboxylate groups connect two metal center ions with different coordination geometries to form a binuclear metal core (Figure 2b).

**Figure 3:**
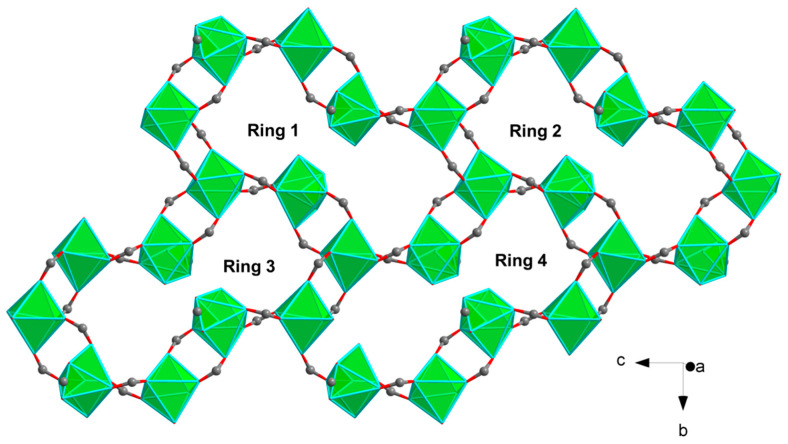
The 2D network structure of compound **1** with ten-membered metal rings along the a-axis.

Interestingly, each metal center is also interconnected with neighboring metal ions via two bridged carboxylic acid groups, with every four head-to-head dimers connected to two neighboring six coordinated Dy2 ions, forming a ten-membered metal ring. Ring 1 and ring 2 and ring 3 and ring 4 are found to have the same configuration, all by sharing two adjacent Dy2 centers. Meanwhile, ring 1 and ring 3 and ring 2 and ring 4 are linked with each other by sharing the edge formed by adjacent Dy2-Dy1-Dy2. In this way, the zero-dimensional cluster unit of the ten-membered metal ring Dy_10_ is further expanded by bridged carboxylic acid groups, giving rise to a two-dimensional network structure along the crystallographic a-axis (Figure 3), which is particularly rare in Ln-CPs.

### 2.3. Magnetic Properties

#### 2.3.1. Static Magnetic Measurements

**Figure 4:**
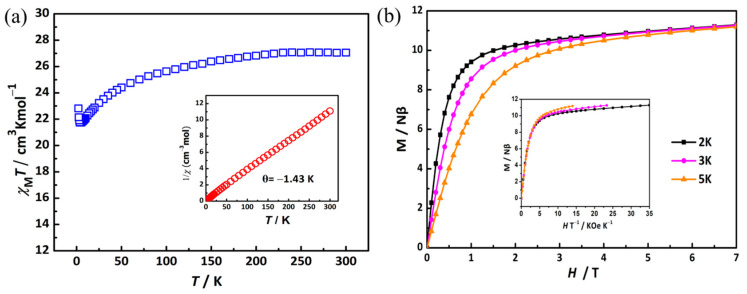
(**a**) Plots of *χ*_M_*T* vs. *T* for compound **1**. (**b**) *M* vs. *H* and *M* vs. *H*/*T* (inset) plots for compound **1** at 2–5 K. The solid lines show a guide for the eyes.

The direct-current (dc) magnetic susceptibility data of compound **1** were collected on polycrystalline samples in the temperature range of 300–2 K under an applied field of 1 KOe as can be seen in Figure 4a. At 300 K, the *χ_M_T* value is 27.31 cm^3^ K mol^−1^, which is close to the desirable value of 28.34 cm^3^ K mol^−1^ for two uncoupled Dy^III^ ions (^6^*H*_15/2_, *S* = 5/2, *L* = 5, and *g* = 4/3). With the temperature cooling, the *χ_M_T* value gradually decreases above 50 K and rapidly decreases to the minimum value of 21.26 cm^3^ K mol^−1^ at around 7 K, and then rapidly increases to 22.75 cm^3^ K mol^−1^ at 2 K. This behavior is probably attributed to the competing ferromagnetic and antiferromagnetic coupling between the Dy^III^ centers [27,28,29]. Meanwhile, the curve of *χ*_M_^−1^ versus *T* obeys the Curie–Weiss law in the range from 300 to 50 K, resulting in Weiss constants being –1.43. The negative Weiss parameter further verifies the existence of weak intramolecular antiferromagnetic interactions.

The isothermal field dependence of magnetization was measured at 2–5 K in the field range of 0–7 T (Figure 4b). The magnetization values increase rapidly from 0 to 2 T, then increase gently from 2 to 7 T, and finally attain the value of 11.28 *Nβ* at 7 T and 2 K, which is prominently smaller than the theoretical saturation value of 20 *Nβ* for two non-interacting Dy^III^ ions. This behavior reveals the presence of significant magnetic anisotropy and/or low-lying excited states [30,31].

#### 2.3.2. Dynamic Magnetic Measurements 

**Figure 5:**
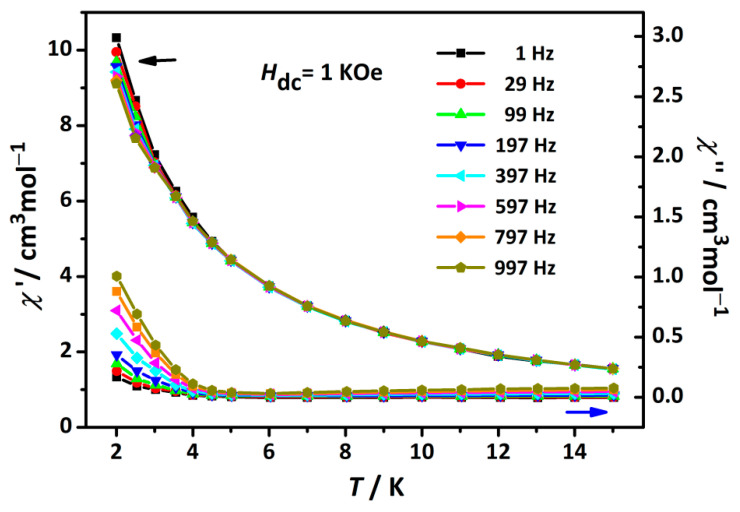
Temperature dependence of the in-phase and out-of-phase ac magnetic susceptibility signals at the indicated frequencies for compound **1** under the optimal dc field.

Considering the significant magnetic anisotropy of Dy^III^ ions, temperature-dependent alternating current (ac) magnetic susceptibility studies were first performed using a 3.5 Oe ac field oscillating at frequencies in the 1–1000 Hz range to explore the possibility of magnetodynamics. As shown in Figure S5, no obvious out-of-phase (*χ*″) signals were found at zero dc field, which might be assigned to the fast quantum tunneling of magnetization (QTM). In order to determine the optimal dc field to suppress the QTM, the ac magnetic susceptibilities of **1** were then measured again in the optimized 1000 dc Oe. Both the temperature- and frequency-dependent out-of-phase (*χ*″) signals were seen but no well-defined maximum peaks were observed (Figure 5 and Figure S5), indicating the slow relaxation of the magnetization behavior characteristic of SMM with a relatively small energy barrier [32,33]. 

### 2.4. Luminescent Properties of Solid-State 2 and H_6_L 

**Figure 6:**
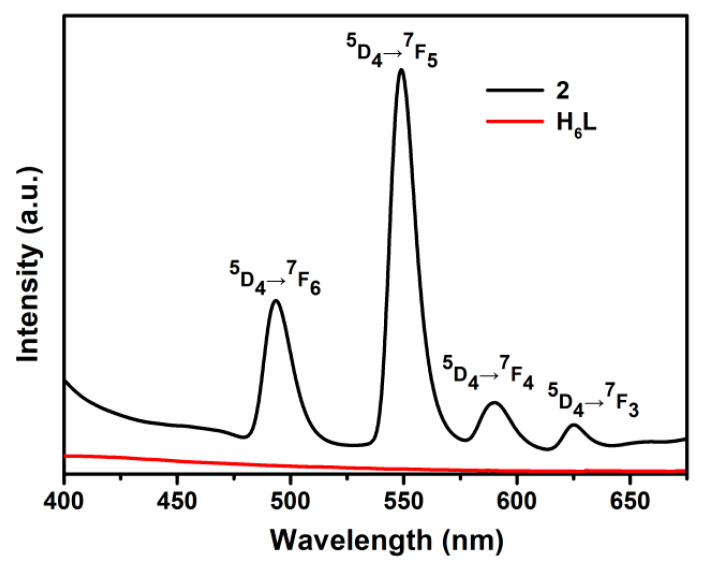
Room temperature solid-state emission spectra of compound **2** and H_6_L under excitation of 352 nm.

Taking into account the excellent luminescent properties of the Tb(III) ion, the luminescent spectra of compound **2** and the ligand H_6_L were investigated in the solid state at room temperature. As shown in Figure 6, H_6_L did not show an obvious fluorescence emission peak. When excited at 360 nm, **2** shows green luminescence with four characteristic emission peaks of Tb^3+^ ions, located at 493 nm, 554 nm, 586 nm, and 627 nm, which may be attributed to the ^5^D_4_→^7^F*_J_* (J = 6, 5, 4, 3) transition of Tb^3+^ ions. The strongest emission peak is located at 554 nm, corresponding to the ^5^D_4_→^7^F_7_ transition. It is worth noting that the ligand has no obvious emission peak, while the fluorescence emission of simple Tb^3+^ is very weak. When the ligand reacts with Tb^3+^ to form compound **2**, a significant solid fluorescence emission peak appears, indicating that the ligand, as an “antenna” [34,35,36], can effectively transmit the energy of absorbed excitation light to metal Tb^3+^ ions. In addition, the excitation spectrum and quantum yield of the ligand H_6_L and compound **2** were also investigated (Figures S7–S10), and the results further prove the occurrence of the antenna effect.

## 3. Materials and Methods

### 3.1. Materials and Measurements

All chemicals were commercially acquired and used directly without further purification unless otherwise specified. Dy(NO_3_)_3_·6H_2_O, Tb(NO_3_)_3_·6H_2_O, N,N-dimethylformamide (DMF), and concentrated HNO_3_ were purchased from Sinopharm Chemical Reagent (Shanghai, China). The hexacarboxylic acid ligand H_6_L was synthesized according to the reported method [13]. Elemental analyses (C, H, and N) were performed on a Perkin-Elmer 2400 analyzer (PerkinElmer, Waltham, MA, USA). Powder X-ray diffraction (PXRD) experiments were carried out at room temperature with an X’pert PRO automatic diffractometer (Cu Ka, Panalytical Company, Almelo, The Netherlands) in the range of 5–50°, and the step size was 0.02° in the 2θ angle. The IR spectra of the synthesized materials were documented in the range of 400–4000 cm^−1^ on a Nicolet 5DX spectrometer (KBr pellets, Thermo Fisher Scientific, Waltham, MA, USA). Thermogravimetric analyses (TGAs) were conducted on a Perkin-Elmer TG-7 thermal analyzer, heated from 25 to 600 °C in a N_2_ atmosphere at a rate of 10 °C/min. Magnetic susceptibility measurements were tested on Quantum Design MPMS XL-7 magnetometer (Quantum Design, San Diego, CA, USA) with a temperature range of 2–300 K and a magnetic field of 1000 Oe.

### 3.2. Synthesis of ***1***

A mixture of hexacarboxylic acid ligand H_6_L (6 mg, 6.26 μmol) and Dy(NO_3_)_3_·6H_2_O (12 mg, 26.31 μmol) was added into the mixed solvent of DMF/H_2_O (*v*/*v*, 4 mL/1 mL) in a 25 mL Teflon-lined stainless steel autoclave. After adding 0.2 mL of 8 M HNO_3_, the sealed autoclave was heated to 130 °C for 3 days, then slowly cooled to room temperature, and colorless block-shaped crystals that were ideal for single-crystal X-ray diffraction were achieved. The crystalline samples were washed with mother liquor, then washed three times with acetone and dried in a vacuum oven at 60 °C (yield 56%, based on H_6_L). Elemental anal. Calcd (%): H 2.37%, C 45.43%, N 4.26%; found H 2.19%, C 45.97%, N 4.30%. FTIR (KBr pellet, cm^−1^): 3454 (br), 3084 (w), 2996 (w), 2671 (w), 2537 (w), 1932 (w), 1699 (s), 1607 (s), 1536 (s), 1423 (s), 1270 (m), 1154 (s), 957 (s), 887 (w), 856 (m), 785 (m), 734 (w), 696 (w).

### 3.3. Synthesis of ***2***

The synthesis procedure of **2** was similar to that of **1** except that Tb(NO_3_)_3_·6H_2_O was used instead of Dy(NO_3_)_3_·6H_2_O; colorless block-shaped crystals suitable for single-crystal X-ray diffraction were obtained (yield 47%, based on H_6_L). Elemental anal. Calcd (%): H 2.37%, C 45.57%, N 4.28%; found H 2.21%, C 45.86%, N 4.52%. FTIR (KBr pellet, cm^−1^): 3454 (br), 3083 (w), 2995 (w), 2670 (w), 2537 (w), 1930 (w), 1699 (s), 1605 (s), 1536 (s), 1423 (s), 1270 (m), 1155 (s), 956 (s), 885 (w), 854 (m), 783 (m), 734 (w), 695 (w).

### 3.4. Single-Crystal X-Ray Diffraction Analysis

The X-ray diffraction data of both complexes were collected at 298 K on a Bruker APEX-II CCD diffractometer (MoK*α* radiation source, *λ* = 0.71073 Å, Bruker, Billerica, MA, USA). The structures of complexes were solved via direct methods with the SHELXT program [37,38] to locate the non-hydrogen atoms from the experimental structures, and then anisotropic refinement was carried out by SHELXTL-2014 using a full-matrix least-squares procedure based on *F*^2^ values. The positions of the hydrogen atoms were geometrically fixed at calculated distances and can ride on the parent atoms. Details of the crystal data and refinement parameters for compounds **1**–**2** are shown in Table 1. The important bond lengths and angles are presented in Tables S1 and S2 (Supporting Information). CCDC 2,384,305 and 2,384,307 include the supplementary crystallographic data for this paper. These data are freely available from The Cambridge Crystallographic Data Centre.

## 4. Conclusions

In conclusion, we have successfully constructed two new lanthanide-based CPs from a flexible cyclotriphosphazene-functionalized hexacarboxylate under the condition of strong acidity using the hydrothermal method. The structures were characterized to be an attractive 2D network structure with ten-membered metal rings linked by bridged carboxyl groups along the crystallographic a-axis. Compound **1** showed weak antiferromagnetic coupling within the metal centers through carboxyl oxygen bridging and behaved as a field-induced SMM, and **2** exhibited attractive luminescence properties. Moreover, in the harsh strong acid synthesis environment, polycarboxylic acid ligands participate in coordination in a semi-protonated state for the first time, which makes these constructed unusual lanthanide-based CPs retain a large number of flexible free carboxyl groups that can interact with metal ions or small organic molecules. Following this lead, more efforts to develop their interesting applications in sensing and adsorption are underway.

## Figures and Tables

**Table 1:** Crystal data and structure refinements for compounds **1**–**2**.

Compound	1	2
Formula	C_87_H_54_ Dy_2_N_7_O_37_P_6_	C_87_H_54_ Tb_2_N_7_O_37_P_6_
*Mr* (g mol^−1^)	2301.2	2294.06
Crystal system	Monoclinic	Monoclinic
Space group	*P*2_1_/c	*P*2_1_/c
*a*/Å	32.278 (7)	32.335 (7)
*b*/Å	16.642 (3)	16.637 (3)
*c*/Å	16.308 (3)	16.305 (3)
*α*/°	90	90
*β*/°	103.09 (3)	103.04 (3)
*γ*/°	90	90
*V*/Å^3^	8533 (3)	8545 (3)
*Z*	4	4
*D*_c_ (g cm^−3^)	1.791	1.783
*μ* (mm^−1^)	1.952	1.855
F(000)	4576	4568
Reflections collected	21,488	14,146
Unique reflections	22,882	15,049
*R_int_*	0.0874	0.1010
Goodness of fit on *F*^2^	1.221	1.026
*R*_1_ *^a^*, *wR*_2_*^b^* [*I* > 2*σ*(*I*)]	0.0878, 0.2456	0.0662, 0.1788
*R*_1_, *wR*_2_ (all data)	0.0899, 0.2524	0.0666, 0.1796
CCDC	2,384,305	2,384,307

*^a^ R*_1_ = ∑||*F*_o_| − |*F*_c_||/∑|*F*_o_|. *^b^ wR*_2_ = [∑*w*(*F*_o_^2^ − *F*_c_^2^)_2_/∑*w*(*F*_o_^2^)_2_]^1/2^.

## Data Availability

Data will be made available upon reasonable request.

## Supplementary Materials

The following supporting information can be downloaded at https://www.mdpi.com/article/10.3390/molecules29235602/s1: Figure S1. FT-IR spectra of compounds **1**–**2**. Figure S2. The simulated X-ray powder diffraction patterns (black) and the experimental ones (red) of compounds **1**–**2**. Figure S3. TGA curve of compounds **1**–**2**. Table S1. Selected bond distances (Å) and angles (°) for **1**. Table S2. Selected bond distances (Å) and angles (°) for **2**. Figure S4. Two different coordination modes of the deprotonated carboxylate groups in hexacarboxylate ligand H_6_L. Figure S5. Temperature dependence of the in-phase (*χ*′) and out-of-phase (*χ*″) ac susceptibility data for **1** in a zero applied dc field. Figure S6. Frequency dependence of the out-of-phase (*χ*″) ac susceptibility data for **1** in a 1000 Oe applied dc field. Figure S7. The excitation spectra of ligand H_6_L (λ_em_ = 440 nm). Figure S8. The excitation spectra of compound **2** (λ_em_ = 545 nm). Figure S9. The quantum yield of ligand H_6_L (λ_em_ = 352 nm). Figure S10. The quantum yield of compound **2** (λ_em_ = 352 nm).
