# Supplementary material for: Synthesis, Structure, and Properties of 2D Lanthanide(III) Coordination Polymers Constructed from Cyclotriphosphazene-Functionlized Hexacarboxylate Ligand
Source: Molecules. 2024 Nov 27;29(23):5602. doi: 10.3390/molecules29235602 (PMC11643244; doi:10.3390/molecules29235602)
Supplement: Supplementary file 1 [file molecules-29-05602-s001.zip › ESI.pdf]

Supporting Information

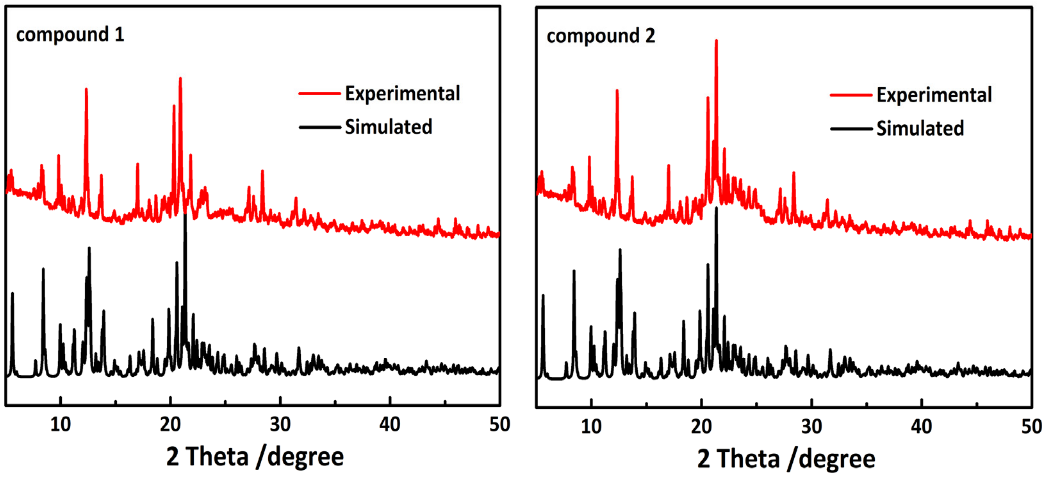

Figure S1. The simulated X-ray powder diffraction patterns (black) and the experimental ones (red) of compounds **1–2**.

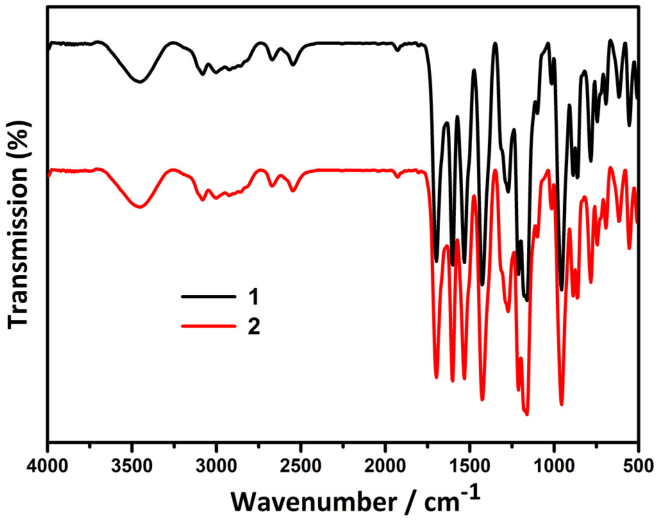

Figure S2. FT-IR spectra of compounds **1–2**.

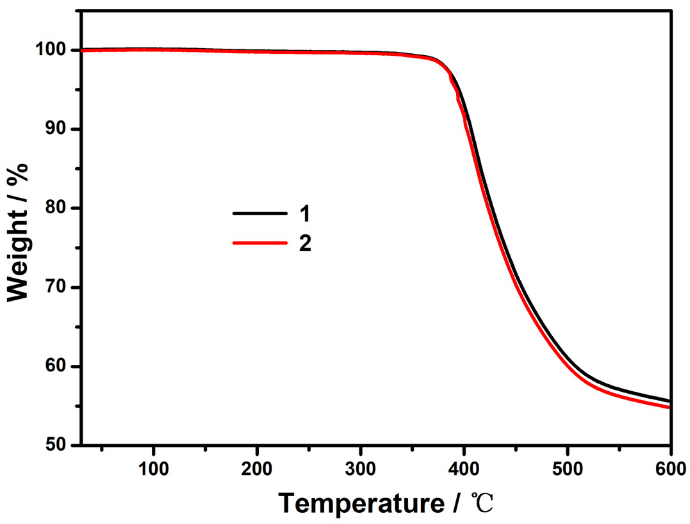

Figure S3. TGA curve of compounds **1–2**.

Table S1. Selected bond distances (Å) and Angles (°) for **1**.

Dy1-O1	2.281(3)	Dy1-O2	2.275(3)
Dy1-O3	2.290(3)	Dy1-O4	2.292(3)
Dy1-O5	2.268(3)	Dy1-O6	2.393(3)
Dy1-O7	2.440(3)	Dy2-O1	2.210(3)
Dy2-O2	2.246(3)	Dy2-O3	2.252(3)
Dy2-O4	2.258(3)	Dy2-O5	2.236(3)
Dy2-O6	2.257(3)	Dy1-Dy2	5.045(9)
Dy1-Dy1	8.926(15)	Dy2-Dy2	9.258(15)
O1-Dy1-O2	75.08(12)	O1-Dy1-O6	87.17(12)
O5-Dy1-O11	98.18(10)	O5-Dy1-O10	107.78(11)
O8- Dy1-O11	125.63(10)	O8-Dy1-O6	95.79(11)
O4-Dy2-O12	81.48(11)	O13-Dy2-O12	99.34(11)
O9-Dy2-O12	96.17(13)	O3-Dy2-O12	83.86(12)
O19-P1-O16	94.53(15)	O15-P2-O18	98.71(15)
O17-P3-O14	97.20(16)	N2-P1-N3	116.13(17)
N3-P2-N4	117.39(17)	N4-P3-N2	117.30(17)

Table S2. Selected bond distances (Å) and Angles (°) for **2**.

Tb1 -O1	2.278(4)	Tb1-O2	2.314(4)
Tb1-O3	2.301(4)	Tb1 -O4	2.294(4)
Tb1-O5	2.280(4)	Tb1-O6	2.449(4)
Tb1-O7	2.400(4)	Tb2-O1	2.273(4)
Tb2-O2	2.257(4)	Tb2-O3	2.272(4)
Tb2-O4	2.263(4)	Tb2-O5	2.223(4)
Tb2-O6	2.261(4)	Tb1- Tb2	5.043(9)
Tb1-Tb1	8.923(15)	Tb2- Tb2	9.267(15)
O1-Tb1-O2	74.62(15)	O1-Tb1-O6	87.41(15)
O5-Tb1-O11	99.04(13)	O5-Tb1-O10	108.56(14)
O8-Tb1-O11	125.13(13)	O8-Tb1-O6	95.38(14)
O4-Tb2-O12	80.08(14)	O13-Tb2-O12	100.84(14)
O9- Tb2-O12	96.72(17)	O3-Tb2-O12	84.32(15)
O19-P1-O16	94.72(18)	O15-P2-O18	98.60(19)
O17-P3-O14	97.00(19)	N2-P1-N3	115.9(2)
N3-P2-N4	117.9(2)	N4-P3-N2	117.7(2)

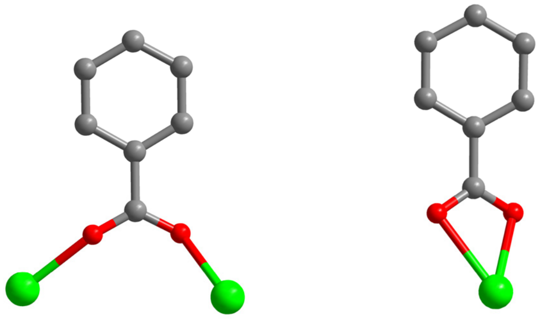

Figure S4. Two different coordination modes of the deprotonated carboxylate groups in hexa-carboxylate ligand H_6L.

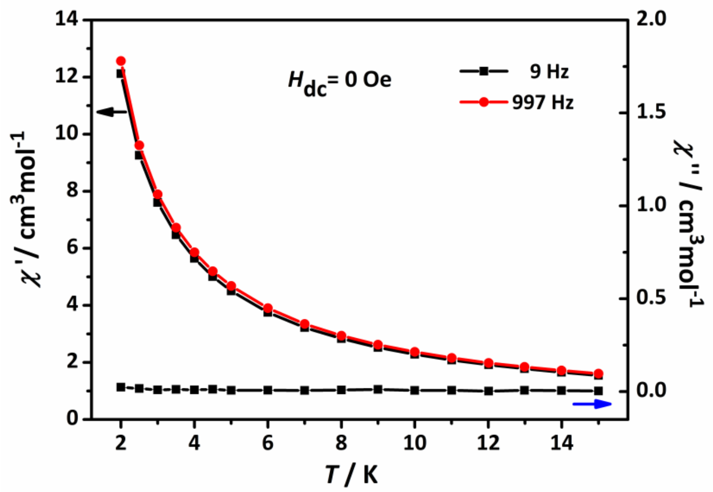

Figure S5. Temperature dependence of the in-phase (χ') and out-of-phase (χ'') ac susceptibility data for **1** under zero applied dc field.

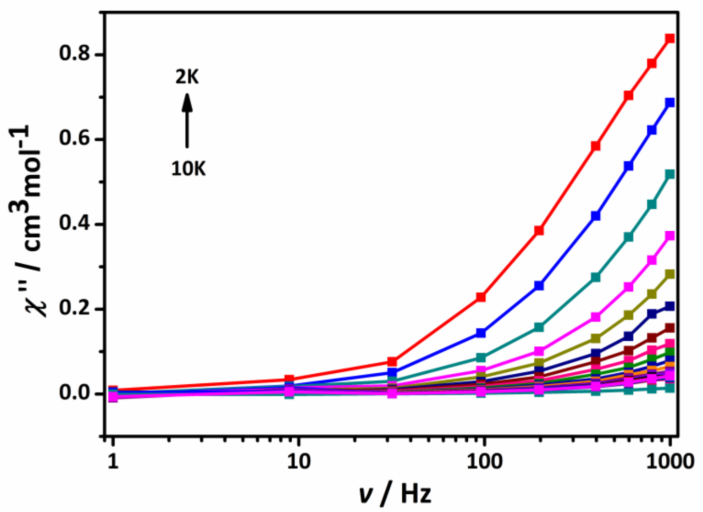

Figure S6. Frequency dependence of the out-of-phase (χ'') ac susceptibility data for **1** under a 1000 Oe applied dc field.

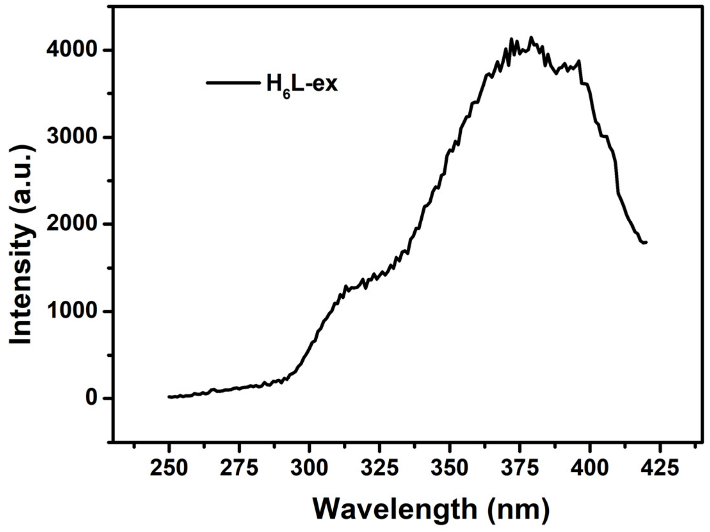

Figure S7. The excitation spectra of ligand H_6L (λ_{em} = 440 nm).

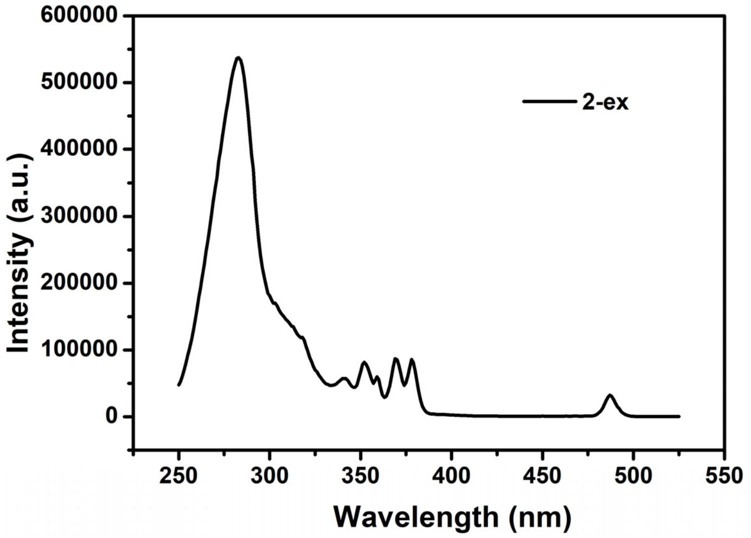

Figure S8. The excitation spectra of compound 2 (λ_{em} = 545 nm).

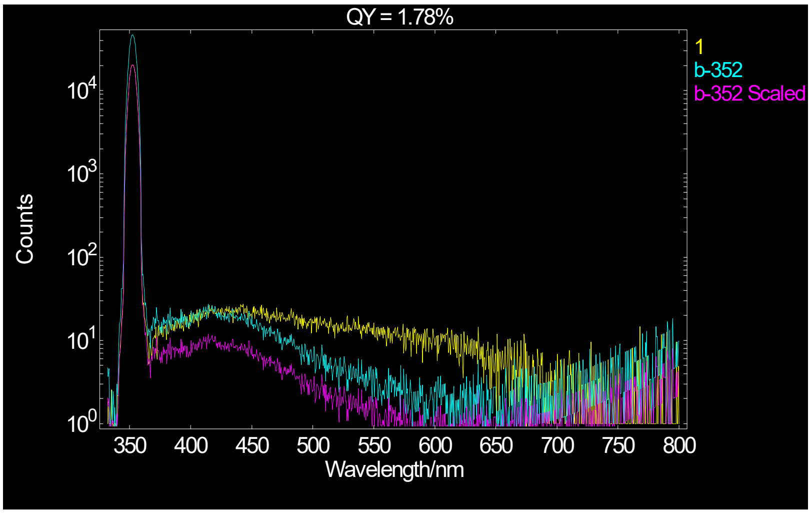

Figure S9. The quantum yield of ligand H_6L (λ_{em} = 352 nm).

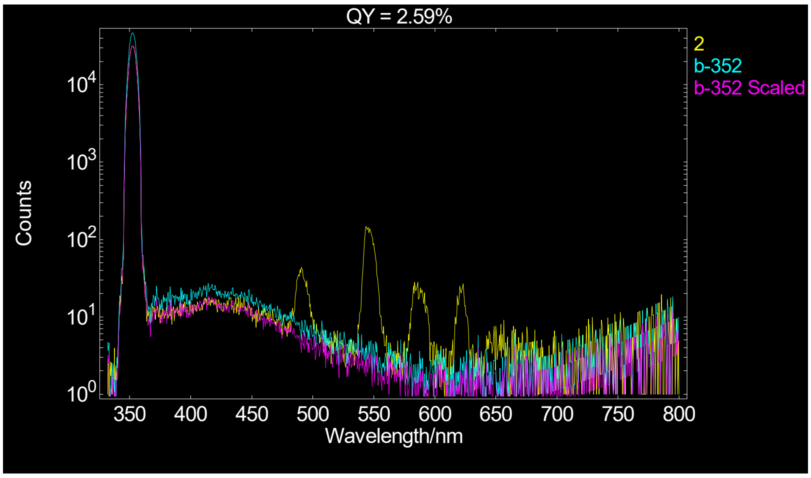

Figure S10. The quantum yield of compound 2 (λ_{em} = 352 nm).
