# Supplementary material for: Synthesis, Structure, and Properties of 2D Lanthanide(III) Coordination Polymers Constructed from Cyclotriphosphazene-Functionlized Hexacarboxylate Ligand
Source: Molecules. 2024 Nov 27;29(23):5602. doi: 10.3390/molecules29235602 (PMC11643244; doi:10.3390/molecules29235602)

checkCIF/PLATON report

You have not supplied any structure factors. As a result the full set of tests cannot be run.

THIS REPORT IS FOR GUIDANCE ONLY. IF USED AS PART OF A REVIEW PROCEDURE FOR PUBLICATION, IT SHOULD NOT REPLACE THE EXPERTISE OF AN EXPERIENCED CRYSTALLOGRAPHIC REFEREE.

No syntax errors found. CIF dictionary Interpreting this report

Datablock: p

```
Bond precision:   C-C = 0.0057 A          Wavelength=0.71073

Cell:              a=32.278(7)     b=16.642(3)        c=16.308(3)
                   alpha=90        beta=103.09(3)     gamma=90
Temperature:       293 K

                 Calculated                  Reported
Volume           8533(3)                     8533(3)
Space group      P 21/c                      P 1 21/c 1
Hall group       -P 2ybc                     -P 2ybc
Moiety formula   C87 H55 Dy2 N7 O37 P6       C87 H55 Dy2 N7 O37 P6
Sum formula      C87 H55 Dy2 N7 O37 P6       C87 H55 Dy2 N7 O37 P6
Mr               2301.20                     2301.20
Dx,g cm-3        1.791                       1.791
Z                4                           4
Mu (mm-1)        1.952                       1.952
F000             4576.0                      4576.0
F000'            4579.19
h,k,lmax         44,22,22                    44,22,22
Nref             22988                       22882
Tmin,Tmax        0.711,0.791
Tmin'            0.697

Correction method= Not given

Data completeness= 0.995          Theta(max)= 29.141

R(reflections)= 0.0874( 21488)                          wR2(reflections)=
                                                        0.2512( 22882)
S = 1.223                 Npar= 1254
```

The following ALERTS were generated. Each ALERT has the format
test-name_ALERT_alert-type_alert-level.
Click on the hyperlinks for more details of the test.

Alert level B

```
PLAT430_ALERT_2_B Short Inter D...A Contact  O20      ..O33      .       2.61 Ang.
                                                    1+x,y,z  =      1_655 Check
```

Author Response: These oxygen atoms are not deprotonated, so hydrogen bonds may be formed between the former and the latter.

```
PLAT430_ALERT_2_B Short Inter D...A Contact  O21      ..O32      .       2.65 Ang.
                                                    1+x,y,z  =      1_655 Check
```

Author Response: These oxygen atoms are not deprotonated, so hydrogen bonds may be formed between the former and the latter.

```
PLAT430_ALERT_2_B Short Inter D...A Contact  O22      ..O34      .       2.56 Ang.
                                                    1+x,y,z  =      1_655 Check
```

Author Response: These oxygen atoms are not deprotonated, so hydrogen bonds may be formed between the former and the latter.

```
PLAT430_ALERT_2_B Short Inter D...A Contact  O23      ..O35      .       2.64 Ang.
                                                    1+x,y,z  =      1_655 Check
```

Author Response: These oxygen atoms are not deprotonated, so hydrogen bonds may be formed between the former and the latter.

```
PLAT430_ALERT_2_B Short Inter D...A Contact  O24      ..O36      .       2.65 Ang.
                                                    1+x,y,z  =      1_655 Check
```

Author Response: These oxygen atoms are not deprotonated, so hydrogen bonds may be formed between the former and the latter.

```
PLAT430_ALERT_2_B Short Inter D...A Contact  O25      ..O37      .       2.64 Ang.
                                                    1+x,y,z  =      1_655 Check
```

Author Response: These oxygen atoms are not deprotonated, so hydrogen bonds may be formed between the former and the latter.

Alert level C

```
PLAT057_ALERT_3_C Correction for Absorption Required   RT(exp) ...       1.11 Do !
```

```
PLAT220_ALERT_2_C NonSolvent   Resd 1  C   Ueq(max)/Ueq(min) Range        4.7 Ratio
PLAT222_ALERT_3_C NonSolvent Resd 1  H   Uiso(max)/Uiso(min) Range        5.5 Ratio
PLAT242_ALERT_2_C Low    'MainMol' Ueq as Compared to Neighbors of        N1 Check
PLAT767_ALERT_4_C INS Embedded LIST 6 Instruction Should be LIST 4     Please Check
```

Alert level G

```
PLAT002_ALERT_2_G Number of Distance or Angle Restraints on AtSite         12 Note
PLAT003_ALERT_2_G Number of Uiso or U(i,j) Restrained non-H Atoms           2 Report
PLAT004_ALERT_5_G Polymeric Structure Found with Maximum Dimension          2 Info
PLAT072_ALERT_2_G SHELXL First  Parameter in WGHT  Unusually Large       0.20 Report
PLAT172_ALERT_4_G The CIF-Embedded .res File Contains DFIX Records         13 Report
PLAT186_ALERT_4_G The CIF-Embedded .res File Contains ISOR Records          1 Report
PLAT199_ALERT_1_G Reported _cell_measurement_temperature ..... (K)        293 Check
PLAT200_ALERT_1_G Reported   _diffrn_ambient_temperature ..... (K)        293 Check
PLAT794_ALERT_5_G Tentative Bond Valency for Dy1       (III)     .       2.99 Info
PLAT794_ALERT_5_G Tentative Bond Valency for Dy2       (III)     .       3.10 Info
PLAT860_ALERT_3_G Number of Least-Squares Restraints ............         25 Note
PLAT883_ALERT_1_G No Info/Value for _atom_sites_solution_primary .     Please Do !
PLAT941_ALERT_3_G Average HKL Measurement Multiplicity ..........         1.0 Low
```

---

0 **ALERT level A** = Most likely a serious problem - resolve or explain
6 **ALERT level B** = A potentially serious problem, consider carefully
5 **ALERT level C** = Check. Ensure it is not caused by an omission or oversight
13 **ALERT level G** = General information/check it is not something unexpected

3 ALERT type 1 CIF construction/syntax error, inconsistent or missing data
11 ALERT type 2 Indicator that the structure model may be wrong or deficient
4 ALERT type 3 Indicator that the structure quality may be low
3 ALERT type 4 Improvement, methodology, query or suggestion
3 ALERT type 5 Informative message, check

It is advisable to attempt to resolve as many as possible of the alerts in all categories. Often the minor alerts point to easily fixed oversights, errors and omissions in your CIF or refinement strategy, so attention to these fine details can be worthwhile. In order to resolve some of the more serious problems it may be necessary to carry out additional measurements or structure refinements. However, the purpose of your study may justify the reported deviations and the more serious of these should normally be commented upon in the discussion or experimental section of a paper or in the "special_details" fields of the CIF. checkCIF was carefully designed to identify outliers and unusual parameters, but every test has its limitations and alerts that are not important in a particular case may appear. Conversely, the absence of alerts does not guarantee there are no aspects of the results needing attention. It is up to the individual to critically assess their own results and, if necessary, seek expert advice.

Publication of your CIF in IUCr journals

A basic structural check has been run on your CIF. These basic checks will be run on all CIFs submitted for publication in IUCr journals (*Acta Crystallographica*, *Journal of Applied Crystallography*, *Journal of Synchrotron Radiation*); however, if you intend to submit to *Acta Crystallographica Section C* or *E* or *IUCrData*, you should make sure that full publication checks are run on the final version of your CIF prior to submission.

Publication of your CIF in other journals

Please refer to the *Notes for Authors* of the relevant journal for any special instructions relating to CIF submission.

PLATON version of 22/08/2024; check.def file version of 21/08/2024

Datablock p - ellipsoid plot

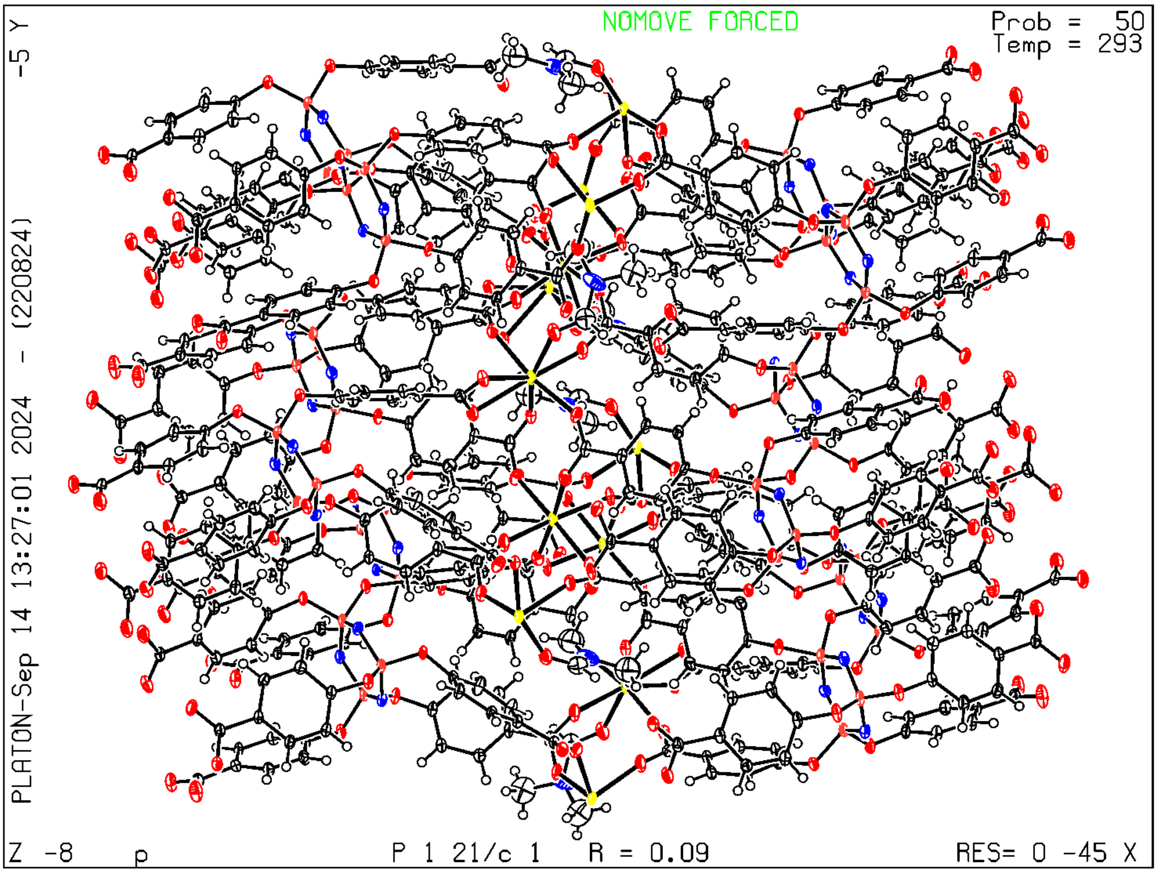

Supplement: Supplementary file 1 [file checkcif-1.pdf › checkcif-1.pdf]
