# Supplementary material for: Synthesis, Structure, and Properties of 2D Lanthanide(III) Coordination Polymers Constructed from Cyclotriphosphazene-Functionlized Hexacarboxylate Ligand
Source: Molecules. 2024 Nov 27;29(23):5602. doi: 10.3390/molecules29235602 (PMC11643244; doi:10.3390/molecules29235602)

checkCIF/PLATON report

You have not supplied any structure factors. As a result the full set of tests cannot be run.

THIS REPORT IS FOR GUIDANCE ONLY. IF USED AS PART OF A REVIEW PROCEDURE FOR PUBLICATION, IT SHOULD NOT REPLACE THE EXPERTISE OF AN EXPERIENCED CRYSTALLOGRAPHIC REFEREE.

No syntax errors found.   CIF dictionary   Interpreting this report

Datablock: p

```
Bond precision:   C-C = 0.0073 A          Wavelength=0.71073

Cell:              a=32.335(7)     b=16.637(3)        c=16.305(3)
                   alpha=90        beta=103.04(3)     gamma=90
Temperature:       293 K

                 Calculated                  Reported
Volume           8545(3)                     8545(3)
Space group      P 21/c                      P 1 21/c 1
Hall group       -P 2ybc                     -P 2ybc
Moiety formula   C87 H55 N7 O37 P6 Tb2       C87 H55 N7 O37 P6 Tb2
Sum formula      C87 H55 N7 O37 P6 Tb2       C87 H55 N7 O37 P6 Tb2
Mr               2294.06                     2294.06
Dx,g cm-3        1.783                       1.783
Z                4                           4
Mu (mm-1)        1.855                       1.855
F000             4568.0                      4568.0
F000'            4571.33
h,k,lmax         38,19,19                    38,19,19
Nref             15049                       15049
Tmin,Tmax        0.766,0.831                 0.765,0.831
Tmin'            0.757

Correction method= # Reported T Limits: Tmin=0.765 Tmax=0.831
AbsCorr = MULTI-SCAN

Data completeness= 1.000          Theta(max)= 24.999

                                                wR2(reflections)=
R(reflections)= 0.0662( 14146)                  0.1796( 14420)

S = 1.026                  Npar= 1254
```

The following ALERTS were generated. Each ALERT has the format
test-name_ALERT_alert-type_alert-level.
Click on the hyperlinks for more details of the test.

Alert level B

PLAT029_ALERT_3_B _diffrn_measured_fraction_theta_full value Low . 0.958 Why?

Author Response: Crystal quality is good, but the diffraction is slightly weak. Many attempts have been made, but this is the best data available.

PLAT430_ALERT_2_B Short Inter D...A Contact O14 ..O32 . 2.56 Ang.
1+x,y,z = 1_655 Check

Author Response: These oxygen atoms are not deprotonated, so hydrogen bonds may be formed between the former and the latter.

PLAT430_ALERT_2_B Short Inter D...A Contact O15 ..O33 . 2.64 Ang.
1+x,y,z = 1_655 Check

Author Response: These oxygen atoms are not deprotonated, so hydrogen bonds may be formed between the former and the latter.

PLAT430_ALERT_2_B Short Inter D...A Contact O16 ..O34 . 2.63 Ang.
1+x,y,z = 1_655 Check

Author Response: These oxygen atoms are not deprotonated, so hydrogen bonds may be formed between the former and the latter.

PLAT430_ALERT_2_B Short Inter D...A Contact O17 ..O35 . 2.60 Ang.
1+x,y,z = 1_655 Check

Author Response: These oxygen atoms are not deprotonated, so hydrogen bonds may be formed between the former and the latter.

PLAT430_ALERT_2_B Short Inter D...A Contact O18 ..O36 . 2.64 Ang.
1+x,y,z = 1_655 Check

Author Response: These oxygen atoms are not deprotonated, so hydrogen bonds may be formed between the former and the latter.

PLAT430_ALERT_2_B Short Inter D...A Contact O19 ..O37 . 2.63 Ang.
1+x,y,z = 1_655 Check

Author Response: These oxygen atoms are not deprotonated, so hydrogen bonds may be formed between the former and the latter.

Alert level C

```
ABSTY02_ALERT_1_C  An _exptl_absorpt_correction_type has been given without
            a literature citation. This should be contained in the
            _exptl_absorpt_process_details field.
            Absorption correction given as multi-scan
PLAT213_ALERT_2_C Atom O3              has ADP max/min Ratio .....        3.3 prolat
PLAT213_ALERT_2_C Atom O20             has ADP max/min Ratio .....        3.6 prolat
PLAT220_ALERT_2_C NonSolvent   Resd 1  C   Ueq(max)/Ueq(min) Range        5.6 Ratio
PLAT222_ALERT_3_C NonSolvent Resd 1  H   Uiso(max)/Uiso(min) Range        6.7 Ratio
PLAT242_ALERT_2_C Low    'MainMol' Ueq as Compared to Neighbors of         N1 Check
PLAT309_ALERT_2_C Single Bonded Oxygen (C-O > 1.3 Ang) ..........         O19 Check
PLAT309_ALERT_2_C Single Bonded Oxygen (C-O > 1.3 Ang) ..........         O36 Check
PLAT369_ALERT_2_C Long   C(sp2)-C(sp2) Bond  C11      - C12      .       1.53 Ang.
```

Alert level G

```
PLAT002_ALERT_2_G Number of Distance or Angle Restraints on AtSite         12 Note
PLAT003_ALERT_2_G Number of Uiso or U(i,j) Restrained non-H Atoms          87 Report
PLAT004_ALERT_5_G Polymeric Structure Found with Maximum Dimension          2 Info
PLAT012_ALERT_1_G No       _shelx_res_checksum Found in CIF ......    Please Check
PLAT066_ALERT_1_G Predicted and Reported Tmin&Tmax Range Identical          ? Check
PLAT072_ALERT_2_G SHELXL First  Parameter in WGHT  Unusually Large       0.12 Report
PLAT083_ALERT_2_G SHELXL Second Parameter in WGHT  Unusually Large      34.16 Why ?
PLAT172_ALERT_4_G The CIF-Embedded .res File Contains DFIX Records          3 Report
PLAT178_ALERT_4_G The CIF-Embedded .res File Contains SIMU Records          1 Report
PLAT186_ALERT_4_G The CIF-Embedded .res File Contains ISOR Records          2 Report
PLAT188_ALERT_3_G A Non-default SIMU Restraint Value has been used     0.0050 Report
PLAT199_ALERT_1_G Reported _cell_measurement_temperature ..... (K)        293 Check
PLAT200_ALERT_1_G Reported   _diffrn_ambient_temperature ..... (K)        293 Check
PLAT794_ALERT_5_G Tentative Bond Valency for Tb1       (III)     .       3.16 Info
PLAT794_ALERT_5_G Tentative Bond Valency for Tb2       (III)     .       3.26 Info
PLAT860_ALERT_3_G Number of Least-Squares Restraints ............        1808 Note
PLAT933_ALERT_2_G Number of HKL-OMIT Records in Embedded .res File          9 Note
              -11  1  9, -14  0  2,   6  5  5,  -3  5  1,  28  0  2,  17  0  0,
               -6  1  2,   5  0  0,  -4  2  7,
PLAT941_ALERT_3_G Average HKL Measurement Multiplicity ..........         2.0 Low
PLAT967_ALERT_5_G Note: Two-Theta Cutoff Value in Embedded .res ..       50.0 Degree
```

```
   0 ALERT level A = Most likely a serious problem - resolve or explain
   7 ALERT level B = A potentially serious problem, consider carefully
   9 ALERT level C = Check. Ensure it is not caused by an omission or oversight
  19 ALERT level G = General information/check it is not something unexpected

   5 ALERT type 1 CIF construction/syntax error, inconsistent or missing data
  18 ALERT type 2 Indicator that the structure model may be wrong or deficient
   5 ALERT type 3 Indicator that the structure quality may be low
   3 ALERT type 4 Improvement, methodology, query or suggestion
   4 ALERT type 5 Informative message, check
```

It is advisable to attempt to resolve as many as possible of the alerts in all categories. Often the minor alerts point to easily fixed oversights, errors and omissions in your CIF or refinement strategy, so attention to these fine details can be worthwhile. In order to resolve some of the more serious problems it may be necessary to carry out additional measurements or structure refinements. However, the purpose of your study may justify the reported deviations and the more serious of these should normally be commented upon in the discussion or experimental section of a paper or in the "special_details" fields of the CIF. checkCIF was carefully designed to identify outliers and unusual parameters, but every test has its limitations and alerts that are not important in a particular case may appear. Conversely, the absence of alerts does not guarantee there are no aspects of the results needing attention. It is up to the individual to critically assess their own results and, if necessary, seek expert advice.

Publication of your CIF in IUCr journals

A basic structural check has been run on your CIF. These basic checks will be run on all CIFs submitted for publication in IUCr journals (*Acta Crystallographica*, *Journal of Applied Crystallography*, *Journal of Synchrotron Radiation*); however, if you intend to submit to *Acta Crystallographica Section C* or *E* or *IUCrData*, you should make sure that full publication checks are run on the final version of your CIF prior to submission.

Publication of your CIF in other journals

Please refer to the *Notes for Authors* of the relevant journal for any special instructions relating to CIF submission.

PLATON version of 22/08/2024; check.def file version of 21/08/2024

Datablock p - ellipsoid plot

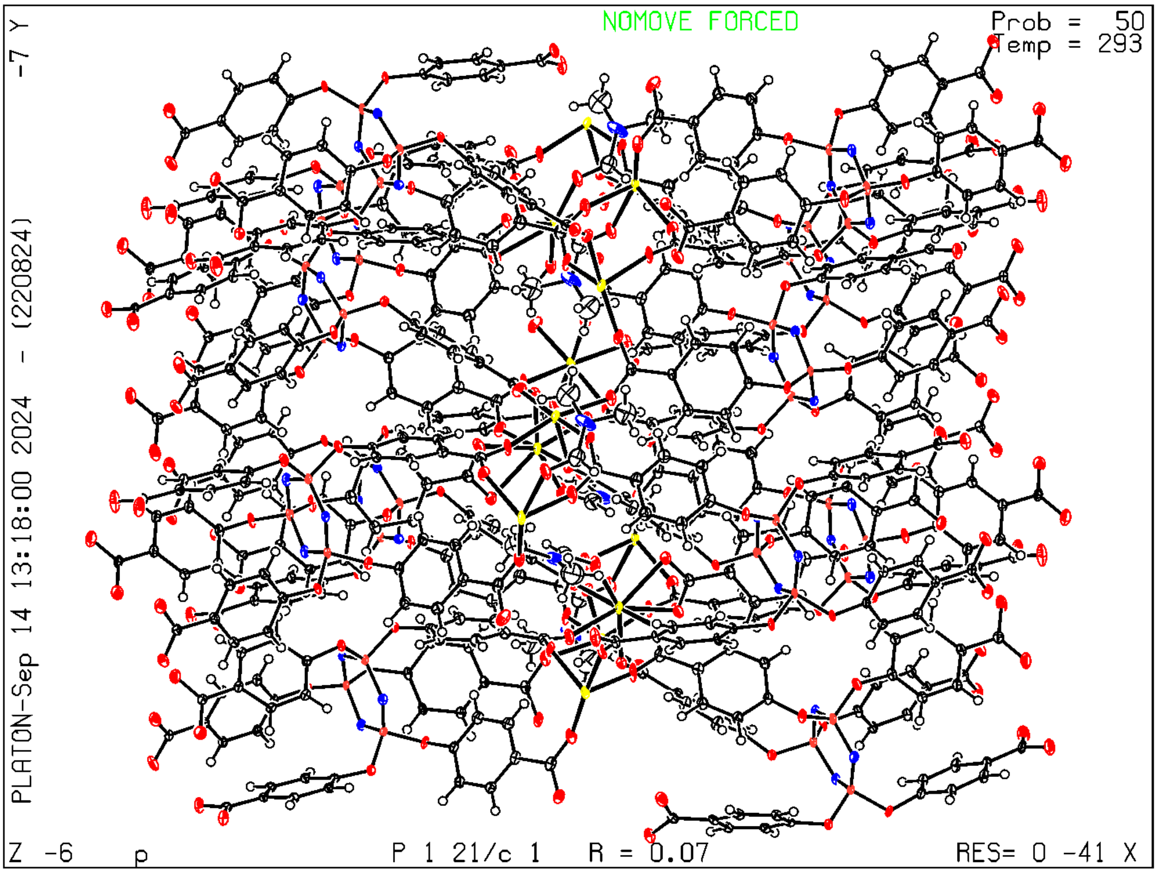

Supplement: Supplementary file 1 [file checkcif-2.pdf › checkcif-2.pdf]
